# Recent and current low food intake – prevalence and associated factors in hospital patients from different medical specialities

**DOI:** 10.1038/s41430-022-01129-y

**Published:** 2022-04-11

**Authors:** Sarah Elisabeth Jasmin Böhne, Michael Hiesmayr, Isabella Sulz, Silvia Tarantino, Rainer Wirth, Dorothee Volkert

**Affiliations:** 1 Institute for Biomedicine of Ageing, Chair of Internal Medicine V, Friedrich-Alexander-Universität Erlangen-Nürnberg, Kobergerstraße 60, 90408 Nuremberg, Germany; 2 Institute for Medical Statistics, Informatics and Intelligent Systems, Medical University of Vienna, Spitalgasse 23, 1090 Vienna, Austria; 3 Department of Geriatric Medicine, Marien Hospital Herne, Ruhr-Universität Bochum, Hölkeskampring 40, 44625 Herne, Germany

**Keywords:** Epidemiology, Risk factors

## Abstract

**Background/Objectives:**

Poor food intake is a major etiological factor of malnutrition. This research aims to describe the prevalence of recent and current low food intake (LI_RC_) and to identify factors associated with LI_RC_ in adult hospital patients from different medical specialities.

**Subject/Methods:**

1865 patients participating in the nutritionDay survey 2016–2020 in Germany were included. LI_RC_ was defined by decreased eating both on nutritionDay and in the week before hospitalisation. Multivariate binary logistic regression was used to identify factors associated with LI_RC_ overall and in different specialities.

**Results:**

LI_RC_ was observed in 21.1% of all patients, with the highest prevalence in Gastroenterology (26.6%) and the lowest in Neurology (11.2%). Weight loss within three months before nutritionDay (OR 2.62 [95% CI 1.93–3.56]), (very) poor self-rated health (2.17 [1.62–2.91]), female sex (1.98 [1.50–2.61]), uncertain weight loss (1.90 [1.03–3.51]), digestive disease (1.90 [1.40–2.56]), inability to walk without assistance (1.55 [1.14–2.12]) and emergency admission (1.38 [1.02–1.86]) were associated with increased risk, cardiac insufficiency (0.55 [0.37–0.83]) and being in a neurological ward (0.51 [0.28–0.92]) with decreased risk in the total sample. In Gastroenterology and Oncology, estimates were higher than in the entire sample; no significant associations were found in Neurology and Geriatrics, presumably due to the low prevalence of LI_RC_ in Neurology and limited data quality in Geriatrics.

**Conclusion:**

LI_RC_ is common in German hospital patients and associated with female sex, poor health and decreased functional status. Interdisciplinary differences suggest a discipline-specific approach to dealing with malnutrition.

## Introduction

Malnutrition is known to impair many organ systems and physiological functions, resulting in poor clinical outcomes [1], increased complication rates, prolonged hospital stays and subsequent higher healthcare costs [2]. Low food intake is a major contributing factor to malnutrition [3] and has been identified as a risk factor for 30-day-mortality through the nutritionDay project [4], an annual worldwide study initiated to increase awareness of disease-related malnutrition in healthcare facilities. Causes of reduced eating are numerous and include disease-related factors (e.g. loss of appetite, feeling too sick or tired), hospital-related (e.g. unfamiliar serving times, meal interruptions) and individual factors (e.g. physical disabilities, need of assistance) [5, 6]. Low food intake during the week before the nutritionDay survey has been associated with reduced eating on nutritionDay [7]. Recent and current low food intake (LI_RC_) both on nutritionDay and prior to hospitalisation may be even more relevant for the development of malnutrition and poor outcome than reduced eating at one single point in time only. To the best of our knowledge, no information about LI_RC_ has been published up to now.

Apart from that, varying malnutrition prevalence rates have been reported in different medical disciplines. According to the Malnutrition Screening Tool (MST), especially patients from oncological, long-term care and infectious disease units are at nutritional risk [8]. Another study using Subjective Global Assessment (SGA) found a significantly higher risk for malnutrition in oncological and gastroenterological patients than in patients from other specialities [9]. Henriksen et al. [10] focused on reduced food intake during the week prior to nutritionDay, and reported that surgical patients were more frequently affected than others. There is, however, no detailed comparison of medical specialities regarding low food intake and associated factors. Knowledge on discipline-related variations could be helpful in counteracting poor eating in a targeted, discipline-specific manner.

Moreover, to date little is known about malnutrition in German hospitals. In a multicentre study from 2006, 27% of the patients were malnourished according to SGA, with wide variation between medical specialities: prevalence ranged from below 10% in Gynaecology to >50% in geriatric patients [11]. Unfortunately, no further up-to-date information has been published despite strong political interest and available nutritionDay data from Germany. Thus, the German nutritionDay database was used with the aim of describing the prevalence of and factors associated with LI_RC_, i.e. both on nutritionDay and in the week before hospital admission, overall and in different medical disciplines.

## Material & methods

### The nutritionDay survey

nutritionDay is an annual one-day cross-sectional study in hospitals, intensive care units and nursing homes worldwide. The project was founded in 2006 with the support of the European Society for Clinical Nutrition and Metabolism (ESPEN) and the Medical University of Vienna in order to raise attention to malnutrition. Participation is open to any interested institution and free of charge, and registration is accomplished online (www.nutritionday.org). The data is collected and entered into the database by local unit staff by means of a standardised questionnaire, which can be downloaded in >30 languages from the nutritionDay website. The questionnaire consists of three parts: one hospital sheet, two unit sheets and four sheets concerning the patient – two filled out by unit staff and two completed by the patients themselves. Hospital patient outcome is collected 30 days after nutritionDay. The survey is approved yearly by the ethics committee of the Medical University of Vienna (number 407/2005) and was also approved by the ethics committee of the Friedrich-Alexander-Universität Erlangen-Nürnberg in Germany in 2018 (number 208_18 B).

### Study participants

For this research project, adult patients (≥18 years) from German hospital units participating in the nutritionDay surveys from 2016 to 2019 and from three additional units participating in August 2020 were included. Patients who participated in the nutritionDay hospital express survey were excluded because of the reduced questionnaire. Patients who did not give oral or written consent, with missing information about speciality and sex, and in wards reporting outcome 30 days after nutritionDay from less than 75% of the participating patients.

### Variables

#### Food intake

LI_RC_ was defined by two variables from the questionnaire filled out by the patient: ‘Lunch eaten on nutritionDay’, described in words and by a symbolic plate used to visualise the eaten meal, was categorised as follows: ‘About all’ was considered normal eating, while ‘1/2’, ‘1/4’ and ‘nothing’ were summarised as reduced intake. The amount of food eaten before hospital admission (‘eaten before admitted’) was defined adequate when patients answered ‘More than normal’ or ‘normal’, and reduced when ‘3/4 of normal’, ‘about half’ or ‘about a quarter to nearly nothing’ were indicated. If patients were unable to complete the questionnaire, data was collected by unit staff. Food intake was defined as recently and currently low (LI_RC_) if it was reduced on nutritionDay as well as in the week before hospital admission.

#### Potential factors associated with *LI*_*RC*_

Medical specialities, specified on the unit questionnaire, were categorised into the following groups: Internal Medicine (Cardiology, Nephrology, Infectious diseases and General Internal Medicine), Gastroenterology (including Hepatology), Geriatrics, Oncology (including Radiotherapy), Surgery (General, Cardiac/Vascular/Thoracic, Orthopaedic, Neurosurgery, Trauma) and Neurology. All other units (Interdisciplinary, Ear Nose Throat, Gynaecology/Obstetrics, Psychiatry, Paediatrics, Others) were summarised as ‘Others’.

From the patient’s questionnaire completed by unit staff, the following variables were considered: age (dichotomised as ‘70 years or older’ and ‘below 70 years’), sex (female – male), body mass index (BMI, calculated as weight/height^2^ and classified into <20, 20–30 and >30 kg/m^2^), admission type (emergency – planned – I do not know), length of hospital stay before nutritionDay (LOS, dichotomised as ‘up to 4 days’ – ‘more than 4 days’), number of medications on nutritionDay (oral and other, categorised into ‘up to 5’ – ‘more than 5’), previous surgery (Yes, planned – Yes, acute – No, categorised into ‘Yes’ and ‘No’), prior ICU admission (Yes – No) and terminally ill (Yes – No – I do not know). ‘Digestive disease’ and ‘endocrine, nutritional and metabolic disease’ were rated as present if chosen by unit staff out of 21 admission diagnoses (multiple answer options), or if indicated as main diagnosis. The following comorbidities were included: cancer, dementia, cardiac insufficiency, infection, and chronic liver, lung and kidney disease (Yes – No, respectively).

From the questionnaire completed by the patients, the subsequent variables were included: weight loss within the three months before nutritionDay (Yes, intentional – Yes, unintentional – No, gained weight – No, stayed the same – I do not know, categorised into ‘Yes’ – ‘No’ – ‘unknown’) and the ability to walk without assistance on nutritionDay (Yes – ‘No, only with assistance’ and ‘No, I stay in bed’ merged into ‘No’). Self-rated general health was asked in five categories (very good – good – fair – poor – very poor) and divided into the two groups ‘fair or better’ and ‘poor or very poor’. Patients who were unable to complete their questionnaire received help from unit staff.

### Statistical analysis

Statistical analysis was performed using IBM SPSS Statistics version 26.0. Absolute frequencies and percentage were calculated for categorical variables, and mean with standard deviation for continuous variables. Univariate binary logistic regression models with LI_RC_ as dependent variable were calculated in the entire sample for each of the above-mentioned variables. Continuous independent variables were categorised for simplicity. The reference group of LI_RC_ consisted of patients with normal food intake both before hospitalisation and on nutritionDay, as well as patients who reported reduced eating at either point in time but not both. Cases with missing information about LI_RC_ were excluded. Participants with missing information on independent variables were also excluded if the percentage of missing values was below 10% in the whole sample. If the percentage was 10% or more, a separate category was created. All univariate models not per se containing either age or sex were adjusted for both variables. A *p* value below 0.1 was set as inclusion criterion for the multivariate binary logistic regression model, which was calculated in the total population. To check for peculiarities in medical specialities, the multivariate model was then calculated separately for every discipline. Odds ratios (OR) with 95% confidence intervals (CI) are reported. Regression models were tested for significance through omnibus chi^2^ test, and effect size is presented according to Cohen’s f^2^.

## Results

### Study participants

**Fig. 1:**
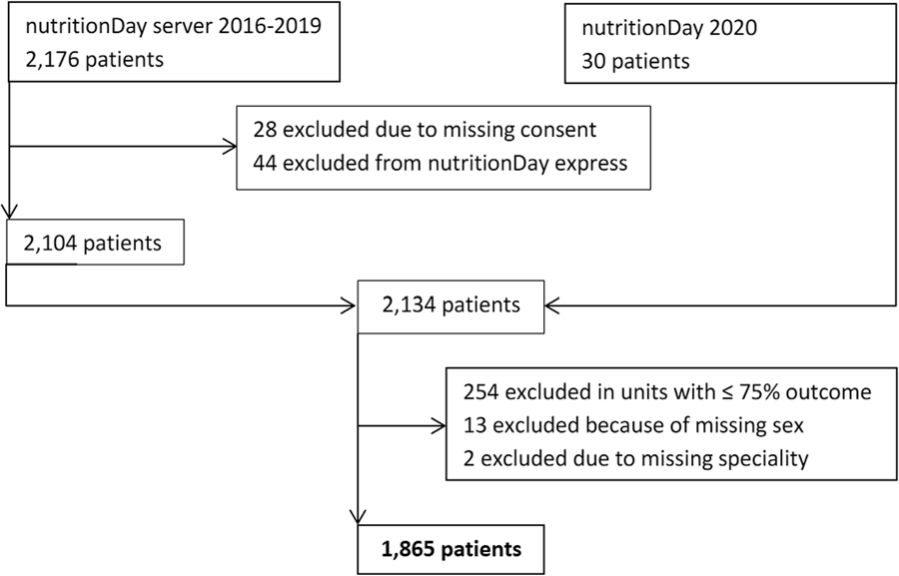
Patient selection process.

The patient inclusion process is described in Fig. 1. 15.5% of patients had to be excluded; mainly to meet the quality criterion of more than 75% completed outcome sheets in the unit. In total, 1865 patients from 127 units in 44 hospitals were included, 23.2% of which from gastroenterological/hepatological units, 17.3% surgical (13.9% General and 3.4% Orthopaedic Surgery) and 15.0% oncological patients. 13.0% were treated in neurological, 11.4% in Internal Medicine (10.7% General Internal Medicine and 0.7% Infectious disease units) and 9.2% in geriatric wards. The group of ‘Others’ (11.0%) was composed of 4.5% interdisciplinary wards, 1.0% from Gynaecology incl. Obstetrics, 0.5% from Otolaryngology and 5.1% from other unspecified specialities.

Table 1 shows characteristics of the patients in the entire sample and according to medical speciality. Mean age in the total population was 67 ± 17 years, and patients were oldest in Geriatric units (83 ± 7 years). Mean body mass index (BMI) was 26 ± 6 kg/m^2^ in the whole sample. A BMI below 20 kg/m^2^ was most frequent in the group of ‘Others’ (14.6%) and least frequent in Neurology (7.4%), which was also the discipline with the highest frequency of obesity (24.8% with BMI > 30 kg/m^2^). Half of all patients reported that they have lost weight within the three months prior to nutritionDay. Neurology stands out with only 36.4%, Internal Medicine with 55.7% and Geriatrics with 55.0% of patients with weight loss history. When taking a closer look, weight loss was mostly reported as unintentional (41.2% vs. 8.7% intentional) in the total population, with 31.1% of all patients having lost >5% of their body weight, and 16.9% losing >10% of their body weight. 46.1% of all admissions were emergencies, nearly two thirds in Internal Medicine wards and less than one third in Oncology units. Digestive disease was most frequent in gastroenterological patients (68.8%) but also very common in other specialities. Endocrine, nutritional or metabolic disease was reported in 24.0% of all cases. Poor or very poor self-rated general health was indicated by 27.5% of patients, most often by geriatric and least often by surgical patients. About two thirds of all patients were able to walk without assistance on nutritionDay, in Geriatrics only about one third.

**Table 1:** Characteristics of patients from the German nutritionDay survey 2016–2020 in the total sample and in different medical specialities.

		Total sample	Internal Medicine	Gastro-enterology	Geriatrics	Oncology	Surgery	Neurology	Others
		*N* = 1865	*n* = 212	*n* = 432	*n* = 171	*n* = 279	*n* = 323	*n* = 242	*n* = 206
		%	%	%	%	%	%	%	%
Age [years]	≥70	49.3	58.5	44.0	97.1	38.7	41.8	41.7	46.1
	<70	50.7	41.5	56.0	2.9	61.3	58.2	58.3	53.9
Sex	Female	51.8	50.9	50.9	69.0	42.3	51.7	58.7	45.6
	Male	48.2	49.1	49.1	31.0	57.7	48.3	41.3	54.4
BMI [kg/m^2^]	<20	10.9	12.3	12.0	12.3	10.4	8.4	7.4	14.6
	20–30	62.5	62.7	61.8	49.7	71.0	62.2	64.9	60.7
	>30	19.5	17.5	20.1	19.9	15.4	21.4	24.8	16.5
	Missing	7.1	7.5	6.0	18.1	3.2	8.0	2.9	8.3
Weight loss within the three months before nDay	Yes	49.9	55.7	52.8	55.0	54.1	48.6	36.4	46.1
	No	39.8	37.3	36.1	28.1	38.7	38.7	56.6	43.7
	Unknown	5.7	4.2	6.5	8.8	4.3	5.6	4.1	7.3
	Missing	4.5	2.8	4.6	8.2	2.9	7.1	2.9	2.9
LOS before nDay [days]	≤4	45.3	42.5	52.8	16.4	47.7	44.9	53.3	44.7
	>4	52.2	53.8	44.9	81.3	50.9	51.7	45.5	51.9
	Missing	2.5	3.8	2.3	2.3	1.4	3.4	1.2	3.4
Admission type	Emergency	46.1	64.6	50.0	59.6	30.1	39.0	42.6	44.7
	Planned	43.5	28.3	36.3	33.9	63.4	47.7	52.5	37.9
	I do not know/missing	10.4	7.1	13.7	6.4	6.5	13.3	5.0	17.5
Cancer	Yes	27.6	21.2	23.6	14.6	70.6	25.4	6.6	22.8
	No	66.0	77.4	70.1	55.6	28.0	72.8	92.6	63.6
	Missing	6.5	1.4	6.3	29.8	1.4	1.9	0.8	13.6
Dementia	Yes	4.0	4.7	1.9	18.1	1.4	2.5	2.1	4.4
	No	88.3	95.3	91.9	45.6	95.3	93.5	96.3	82.0
	Missing	7.7	0.0	6.3	36.3	3.2	4.0	1.7	13.6
Cardiac insuffiency	Yes	17.8	29.7	15.3	39.8	9.7	18.6	9.9	11.7
	No	76.5	68.9	79.4	38.6	88.9	78.3	88.8	75.2
	Missing	5.7	1.4	5.3	21.6	1.4	3.1	1.2	13.1
Infection	Yes	12.5	23.6	11.3	18.1	14.0	10.8	5.4	7.8
	No	79.8	76.4	81.7	46.2	83.2	85.1	93.4	78.2
	Missing	7.7	0.0	6.9	35.7	2.9	4.0	1.2	14.1
Chronic liver disease	Yes	9.7	11.3	20.8	3.5	7.5	5.3	1.7	9.2
	No	82.9	86.3	73.6	59.6	91.0	91.0	96.3	78.6
	Missing	7.4	2.4	5.6	36.8	1.4	3.7	2.1	12.1
Chronic lung disease	Yes	14.6	38.7	11.6	23.4	12.9	6.5	5.8	14.6
	No	78.4	57.5	82.2	49.1	85.7	89.5	93.0	72.3
	Missing	6.9	3.8	6.3	27.5	1.4	4.0	1.2	13.1
Chronic kidney disease	Yes	14.6	13.2	16.7	32.2	12.2	11.1	6.6	15.0
	No	78.5	85.4	77.3	38.0	84.9	85.4	91.7	72.3
	Missing	6.9	1.4	6.0	29.8	2.9	3.4	1.7	12.6
Digestive disease	Yes	38.5	34.4	68.8	17.5	24.4	51.4	4.1	35.9
	No	61.1	64.6	31.3	82.5	74.9	48.3	95.9	63.1
	Missing	0.4	0.9	0.0	0.0	0.7	0.3	0.0	1.0
Endocrine, nutritional and metabolic disease	Yes	24.0	28.3	31.3	31.0	22.6	18.0	16.1	19.4
	No	75.6	70.8	68.8	69.0	76.7	81.7	83.9	79.6
	Missing	0.4	0.9	0.0	0.0	0.7	0.3	0.0	1.0
Nr. of medica-tions on nDay	>5	50.6	52.8	47.5	66.7	53.0	43.7	47.5	52.4
	≤5	23.1	22.2	27.8	8.8	20.1	26.3	21.9	26.7
	M﻿issing	26.3	25.0	24.8	24.6	26.9	30.0	30.6	20.9
Previous surgery	Yes	20.2	5.2	8.1	7.6	12.5	65.3	3.7	30.6
	No	77.2	92.5	89.6	91.2	82.4	31.0	95.9	67.0
	Missing	2.6	2.4	2.3	1.2	5.0	3.7	0.4	2.4
Prior ICU admission	Yes	10.9	8.0	8.1	5.3	4.7	25.1	9.5	12.6
	No	86.1	91.5	88.4	94.7	91.8	69.7	90.1	81.6
	Missing	3.0	0.5	3.5	0.0	3.6	5.3	0.4	5.8
Terminally ill	Yes	13.8	5.2	19.7	2.9	28.0	3.7	19.4	9.2
	No	64.7	83.5	58.3	74.9	42.7	74.6	72.3	55.8
	I do not know	21.5	11.3	22.0	22.2	29.4	21.7	8.3	35.0
Self-rated general health	Fair or better	68.5	66.5	68.1	59.6	64.9	73.7	69.4	74.3
	Poor or very poor	27.5	31.1	28.0	34.5	30.1	19.8	30.2	21.8
	Missing	4.1	2.4	3.9	5.8	5.0	6.5	0.4	3.9
Ability to walk without assistance on nDay	Yes	62.4	63.7	67.4	34.5	72.8	60.4	54.1	72.3
	No	30.5	29.7	24.1	54.4	22.2	29.7	43.4	21.8
	Missing	7.2	6.6	8.6	11.1	5.0	9.9	2.5	5.8

*ICU* intensive care unit, *LOS before nDay* length of hospital stay before nutritionDay, *nDay* nutritionDay.

### Low food intake

**Fig. 2:**
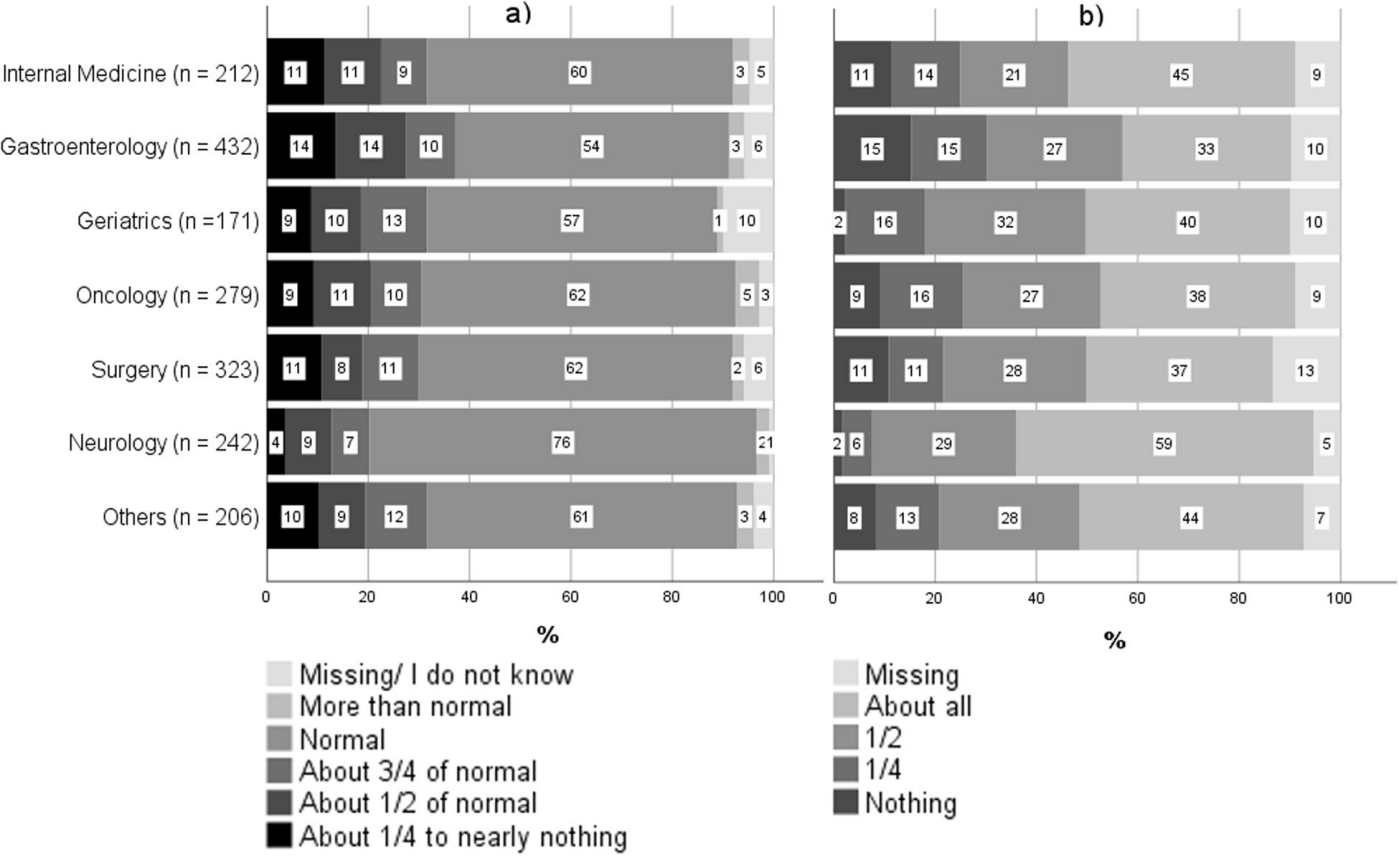
Food intake of patients from the German nutritionDay survey 2016–2020 in different medical specialities. **a** In the week before hospital admission and (**b**) on nutritionDay.

In the week before hospital admission, 31.0% of the patients reported reduced food intake, with Neurology units showing the lowest (20.2%) and Gastroenterology wards the highest (37.3%) prevalence (Fig. 2a). Half of all patients (49.5%) ate only half or less of the served meal on nutritionDay. The highest prevalence was found in Gastroenterology (56.9%), where the percentage of patients eating nothing at all was also the highest (15.3%). Neurological patients most often reported having eaten the full meal (58.7%) (Fig. 2b). The frequency of reduced food intake both on nutritionDay and prior to hospitalisation is presented in Table 2. 21.1% of all patients reported LI_RC_ with Gastroenterology patients leading the field (26.6%). Only 11.2% of neurological patients indicated LI_RC_.

**Table 2:** Prevalence of recent and current low food intake in the total sample and in different medical specialities.

	Total sample	Internal medicine	Gastro-enterology	Geriatrics	Oncology	Surgery	Neurology	Others
	*N* = 1865	*n* = 212	*n* = 432	*n* = 171	*n* = 279	*n* = 323	*n* = 242	*n* = 206
	%	%	%	%	%	%	%	%
Yes	21.1	22.2	26.6	22.8	21.9	19.5	11.2	19.9
No	72.8	73.6	65.0	66.1	74.2	72.4	88.0	74.8
Missing	6.1	4.2	8.3	11.1	3.9	8.0	0.8	5.3

About one quarter of all patients (27.7%) had reduced intake only on nutritionDay but not in the week before admission, and 8.2% reported reduced intake only in the week before admission but not on nutritionDay. Two thirds (68.0%) of those with reduced intake before admission (*n* = 578) also had reduced intake on nutritonDay, and 42.5% of those with reduced intake on nutritionDay (*n* = 924) also had low intake in the week before admission.

### Factors associated with *LI*_*RC*_

In the univariate analyses, the subsequent variables met the selection criterion and were thus included in the multivariate model: age; sex; BMI; weight loss within the three months before nutritionDay; admission type; cardiac insufficiency; infection; digestive disease; endocrine, nutritional or metabolic disease; self-rated general health; ability to walk without assistance on nutritionDay; and medical speciality (Table S1). According to the multivariate logistic regression in the entire sample, female sex, weight loss and unknown weight loss history, emergency admissions, digestive disease, poor or very poor self-rated general health, and not being able to walk without assistance on nutritionDay were positively associated with LI_RC_. Neurology admission and cardiac insufficiency were related to a reduced risk of LI_RC_ (Table 3). Regarding medical specialities, most associations and consistency with the whole sample were found in Gastroenterology and Oncology patients (Table 4). Odds ratios of significant associations were generally higher in the subgroups than in the total population, and highest in Oncology, especially for poor or very poor self-rated general health, unknown admission type and female sex. In Gastroenterology, odds ratios for weight loss and unknown weight loss were about 4- and 3-times increased compared with no weight loss. In the Internal Medicine sample, patients unable to walk without assistance on nutritionDay had the highest risk of LI_RC_, whereas the odds ratio of cardiac insufficiency was reduced. Weight loss and digestive disease were relevant in Surgery and the group of other specialities. No significant associations were found in both Geriatrics and Neurology. All multivariate models except those of Neurology and Geriatrics were significant in the chi^2^ test for overall model fit, and effect size was strong for Internal Medicine, Gastroenterology and Oncology (Cohen’s f^2^ ≥ 0.35), medium for the overall sample, Geriatrics, Surgery and Others (≥0.15), and low for Neurology (≥0.02).

**Table 3:** Multivariate binary logistic regression model: Odds ratios (OR) and 95% confidence intervals (CI) for recent and current low food intake in the total sample (*N* = 1410).

		OR	[CI 95%]
Age [years]	<70 (ref)	1.00	
	≥70	1.17	[0.87–1.57]
Sex	Male (ref)	1.00	
	Female	1.98	[1.50–2.61]***
BMI [kg/m^2^]	<20	1.11	[0.74–1.68]
	20–30 (ref)	1.00	
	>30	1.00	[0.70–1.42]
Weight loss within the three months before nDay	No (ref)	1.00	
	Yes	2.62	[1.93–3.56]***
	Unknown	1.90	[1.03–3.51]*
Admission type	Planned (ref)	1.00	
	Emergency	1.38	[1.02–1.86]*
	I do not know/ Missing	1.41	[0.87–2.29]
Cardiac insufficiency	No (ref)	1.00	
	Yes	0.55	[0.37–0.83]**
Infection	No (ref)	1.00	
	Yes	1.17	[0.80–1.71]
Digestive disease	No (ref)	1.00	
	Yes	1.90	[1.40–2.56]***
Endocrine, nutritional and metabolic disease	No (ref)	1.00	
	Yes	0.81	[0.58–1.11]
Self-rated general health	Fair or better (ref)	1.00	
	Poor or very poor	2.17	[1.62–2.91]***
Ability to walk without assistance on nDay	Yes (ref)	1.00	
	No	1.55	[1.14–2.12]**
Medical speciality	Internal Med.(ref)	1.00	
	Gastro-enterology	1.17	[0.73–1.89]
	Geriatrics	1.01	[0.51–2.01]
	Oncology	1.16	[0.69–1.94]
	Surgery	0.91	[0.55–1.51]
	Neurology	0.51	[0.28–0.92]*
	Others	0.96	[0.53–1.72]
Model significance	*p* value	0.000	
Effect size	Cohen’s f^2^	0.230	

*ref* reference.

*p < 0.05; **p < 0.01; ***p < 0.001.

**Table 4:** Multivariate binary logistic regression models: Odds ratios (OR) and 95% confidence intervals (CI) for recent and current low food intake according to medical speciality.

		Internal medicine		Gastroenterology		Geriatrics		Oncology		Surgery		Neurology		Others	
		*n* = 169		*n* = 327		*n* = 72		*n* = 236		*n* = 247		*n* = 215		*n* = 144	
		OR	[CI 95%]	OR	[CI 95%]	OR	[CI 95%]	OR	[CI 95%]	OR	[CI 95%]	OR	[CI 95%]	OR	[CI 95%]
Age [years]	<70 (ref)	1.00		1.00		1.00		1.00		1.00		1.00		1.00	
	≥70	1.25	[0.48–3.28]	1.20	[0.68–2.12]	1.70	[0.05–58.9]	1.41	[0.65–3.04]	1.17	[0.55–2.49]	1.56	[0.62–3.95]	1.02	[0.39–2.71]
Sex	Male (ref)	1.00		1.00		1.00		1.00		1.00		1.00		1.00	
	Female	1.97	[0.83–4.68]	1.88	[1.08–3.29]*	1.38	[0.32–5.93]	3.77	[1.71–8.30]**	1.47	[0.73–2.96]	2.43	[0.92–6.40]	1.75	[0.68–4.51]
BMI [kg/m^2^]	<20	1.30	[0.41–4.11]	2.00	[0.92–4.35]	2.61	[0.39–17.6]	0.70	[0.22–2.21]	1.29	[0.42–3.99]	0.98	[0.19–5.15]	0.29	[0.07–1.21]
	20–30 (ref)	1.00		1.00		1.00		1.00		1.00		1.00		1.00	
	>30	0.70	[0.22–2.19]	0.82	[0.39–1.70]	1.60	[0.29–8.98]	0.61	[0.21–1.74]	1.57	[0.68–3.61]	1.46	[0.54–3.92]	0.50	[0.13–1.90]
Weight loss in 3 months before nDay	No (ref)	1.00		1.00		1.00		1.00		1.00		1.00		1.00	
	Yes	1.84	[0.75–4.47]	4.41	[2.29–8.52]***	0.67	[0.15–2.96]	3.35	[1.45–7.73]**	3.78	[1.68–8.51]**	1.65	[0.67–4.08]	2.65	[0.89–7.90]
	Unknown	0.00	0.00	3.03	[1.03–8.96]*	2.11	[0.18–24.5]	0.94	[0.14–6.34]	1.08	[0.17–7.09]	2.55	[0.38–16.9]	10.30	[1.54–68.6]*
Admission type	Planned (ref)	1.00		1.00		1.00		1.00		1.00		1.00		1.00	
	Emergency	1.03	[0.38–2.80]	0.97	[0.54–1.76]	3.39	[0.70–16.3]	2.59	[1.20–5.62]*	2.02	[0.94–4.34]	0.92	[0.36–2.37]	1.90	[0.64–5.67]
	I don’t know	1.81	[0.33–9.76]	1.26	[0.53–2.97]	3.47	[0.13–90.3]	5.76	[1.29–25.7]*	1.81	[0.53–6.16]	0.00	0.00	1.61	[0.42–6.16]
Cardiac insufficiency	No (ref)	1.00		1.00		1.00		1.00		1.00		1.00		1.00	
	Yes	0.16	[0.05–0.55]**	0.82	[0.37–1.81]	0.43	[0.10–1.85]	0.49	[0.11–2.21]	0.62	[0.24–1.62]	1.04	[0.25–4.35]	1.12	[0.25–5.01]
Infection	No (ref)	1.00		1.00		1.00		1.00		1.00		1.00		1.00	
	Yes	1.97	[0.74–5.27]	0.54	[0.22–1.33]	1.43	[0.36–5.70]	1.33	[0.52–3.41]	1.26	[0.46–3.46]	0.45	[0.05–4.20]	1.78	[0.42–7.48]
Digestive disease	No (ref)	1.00		1.00		1.00		1.00		1.00		1.00		1.00	
	Yes	1.75	[0.75–4.12]	1.38	[0.76–2.48]	0.81	[0.13–4.93]	1.39	[0.62–3.11]	3.43	[1.64–7.17]**	0.61	[0.06–6.14]	3.85	[1.41–10.5]**
Endocrine, nutr., metab. diseases	No (ref)	1.00		1.00		1.00		1.00		1.00		1.00		1.00	
	Yes	0.87	[0.32–2.38]	0.65	[0.36–1.19]	0.37	[0.09–1.54]	0.52	[0.21–1.33]	1.15	[0.47–2.76]	1.12	[0.35–3.61]	1.37	[0.46–4.11]
Self-rated general health	Fair or better (ref)	1.00		1.00		1.00		1.00		1.00		1.00		1.00	
	Poor or very poor	2.07	[0.84–5.15]	2.23	[1.25–3.97]**	3.17	[0.78–12.9]	6.76	[3.09–14.8]***	1.09	[0.44–2.69]	1.88	[0.74–4.77]	1.73	[0.59–5.12]
Ability to walk without assistance on nDay	Yes (ref)	1.00		1.00		1.00		1.00		1.00		1.00		1.00	
	No	3.11	[1.23–7.87]*	2.17	[1.16–4.05]*	0.43	[0.10–1.82]	1.37	[0.59–3.18]	2.33	[1.06–5.10]*	1.76	[0.66–4.65]	0.47	[0.11–2.00]
Model significance	*p* value	0.001		0.000		0.635		0.000		0.000		0.389		0.045	
Effect size	Cohen’s f^2^	0.395		0.361		0.279		0.484		0.330		0.147		0.316	

*nDay* nutritionDay, *ref* reference.

*p < 0.05; **p < 0.01; ***p < 0.001.

## Discussion

### Low food intake

This analysis of recent data from German hospitals participating in the nutritionDay study focuses on reduced nutritional intake as an important etiological contributor to malnutrition [3], which is associated with poor outcomes [4]. Low food intake at a single meal on nutritionDay was examined earlier, and prevalence rates of 53% [7] and 52% [4] were reported in worldwide samples. In this analysis, also about half of the patients reported low food intake on nutritionDay, thereby confirming that this problem is found to a similar extent in German hospitals, despite the rather good health care system. When focusing only on one meal at the hospital, however, this potentially includes patients with good nutrition who did not like the hospital food and had visitors bring food from home, as well as patients who normally eat smaller portions, who missed the meal because of a medical procedure, or who did not eat up for some other external reason [12]. By contrast, this research aims to give a more comprehensive picture of low food intake by considering nutritional intake prior to hospitalisation in addition to the amount eaten on nutritionDay. It can be assumed that patients with LI_RC_ already had their health problems some time prior to admission, whereas observation of low food intake only on nutritionDay might rather reflect an acute health problem and acute disease-related malnutrition. In any case, with a prevalence of 21%, LI_RC_ was very common among German hospital patients. Since nearly half of the patients who ate little on nutritionDay had eaten poorly already before hospital admission, it appears reasonable to inquire about previous nutrition at admission to identify patients in need of further support. Moreover, according to an additional question (not yet described), most patients with LI_RC_ reported an unfavourable development of nutritional intake during their hospital stay. 50.9% reported a further decrease, and 22.9% constantly reduced intake between hospital admission and nutritionDay. Only 9.9% of LI_RC_ patients reported increasing food intake during hospitalisation while still not eating the full meal on nutritionDay. These figures underpin the assumption of a longer lasting problem that was also present in the period between the two assessment times.

### Factors associated with *LI*_*RC*_ overall

More than half of the variables (12 out of 22) examined in the univariate analyses of the total sample were included in the multivariate model, eight of which were still associated with LI_RC_ in the multivariate model with similar odds ratios (Table 3). The choice of variables for this analysis was guided by previous research on malnutrition in general [3, 13–19] and food intake specifically [7]. Female sex is a known risk factor for low food intake on nutritionDay, which was explained by women generally eating smaller meal portions and having stronger weight concerns [7]. The connection to LI_RC_ and thus to pre-hospital nutrition, however, supports only the latter. Surprisingly, being aged 70 years and above was not related to LI_RC_, and nor was a BMI of below 20 kg/m^2^. Higher age is generally considered a risk factor [20] and low BMI is an important phenotypic criterion of malnutrition [3]. Nonetheless, categorisation of both variables differed from the previous analysis, where younger (18–29 years and 30–39 years) and older age (80–89 years and above), as well as a BMI below 18.5 kg/m^2^ were significantly associated with low food intake on nutritionDay [7]. Our analysis confirms that weight loss is clearly associated with LI_RC_, implying an even longer than one-week period of low intake before admission in many patients, as weight loss is usually a process which develops over weeks or even months. Yet, the high odds ratio for patients who were not sure about a weight loss should draw health care workers’ attention. In this subgroup, the proportion of patients with LI_RC_ was higher (26.2%) than in the total sample (data not shown). Older and severely ill patients, having lost the sense of their body or not being able to rate their health status, might have lost weight unknowingly because of LI_RC_. Malnutrition in hospitals is often disease-related. Thus, it is not surprising that a poor or very poor self-rated general health is associated with LI_RC_. It also appears logical that patients with affection of the digestive system are more likely to eat little because of related symptoms such as nausea, vomiting or loss of appetite, and because their food intake at the hospital is often restricted by doctors. Interestingly, cancer was not included in the multivariate regression model by the selection criterion used, although it is commonly known to imply nutritional risk [15]. Acute cancer as admission diagnosis has previously been associated with current low intake and might be a plausible reason for LI_RC_, too [7]. Comorbid cancer, however, rather includes patients with former or stable malign disease who did not show a higher risk of LI_RC_ in this analysis. The negative correlation of cardiac insufficiency with LI_RC_ is remarkable and probably partly explained by the finding that a BMI below 20 kg/m^2^ was less frequent, and above 30 kg/m^2^ was more frequent in patients with cardiac insufficiency (data not shown), suggesting a lower likelihood of LI_RC_. Unfortunately, the stage of this disease, a strong factor influencing the development of cardiac cachexia [21], is not known.

### Role of the medical speciality

Pirlich et al. [11] compared the prevalence of malnutrition in German hospital patients from different medical specialities, finding the highest prevalence in Geriatrics, Oncology, Gastroenterology and other medical patients. Interestingly, the most affected specialities were similar in the present analysis, reaffirming the important role of malnutrition in these fields. Results of the speciality-specific multivariate regression models showed a high consistency for significant variables with the overall sample. Intriguingly, odds ratios of significant variables were considerably higher in the relevant subgroups than in the entire population (Table 4), suggesting a discipline-specific importance. In Gastroenterology and Oncology especially, many factors associated with LI_RC_ were confirmed, with the highest odds ratios in Oncology. In contrast, admission to Neurology was associated with reduced risk for LI_RC_ compared with Internal Medicine patients (Table 3). Additionally, LI_RC_ was the least frequent and without significant associations in Neurology, suggesting that low food intake is a rather minor and non-specific problem in this discipline. This analysis partly confirms results from Schindler et al. [7], where patients from Neurology and Geriatrics were at lower risk for reduced food intake on nutritionDay. At first glance, LI_RC_ does not seem to play an important role in Geriatrics either, as no significant associations were found in this subgroup. However, the prevalence of LI_RC_ in Geriatrics was twice as high as in Neurology, suggesting that the lack of significant associations can be explained by the limited data quality in this subgroup, which is mainly due to a high percentage of missing values for comorbidities, and subsequently leads to the small number of 72 patients in this subgroup after exclusion of missing values. The lacking significance of weight loss in Internal Medicine is surprising. Unfortunately, the reasons are unclear and should be addressed in future research.

### Strengths and limitations

The biggest strength and uniqueness of this study was the close look at LI_RC_, reflecting reduced food intake for a longer period of time, which is very plausible to be more relevant for the development of malnutrition than only intake on nutritionDay. As food intake measurements were self-reported by the patients, data might reflect the patients’ individual perception and their estimating abilities. Thus, it might be less reliable than if it was collected by a designated researcher. Further, retrospective data elicitation holds the risk of bias. Patients might not remember their previous food intake correctly, and their perception of current intake at the hospital might influence the appraisal of their recent intake. There is, however, always a certain degree of uncertainty when recording food intake. An additional strength of the present analysis is the focus on one country with the same health care system in all participating hospitals. This analysis of a large and recent German dataset increases our knowledge on low food intake as a contributor to malnutrition in German hospitals, where little is currently known in this regard. Schindler et al. [7] reported wide variations in the prevalence of low intake on nutritionDay between different world regions. Therefore, a country-specific approach seems also reasonable for the assessment of LI_RC_. However, the country-specific focus led to a smaller sample, which is a limitation of this research. The sample was further reduced in the regression analysis due to missing values, caused, for example, by staff untrained in data collection. The identification of potential associations in small samples such as the geriatric group is difficult; hence, further analyses with larger patient groups are desirable. Moreover, length bias is a common limitation of cross-sectional studies, as patients are more likely to be included if they stay longer at the hospital [22]. Selection bias is another possible limitation. The sample may not be representative for the German hospital population, since patients aged 70 years or older were overrepresented (personal communication: German Federal Statistical Office, hospital statistics from 2019, obtained 2021), as well as patients with digestive disease and endocrine, nutritional or metabolic disease [23]. On the other hand, sex distribution was comparable to data reported by German hospital statistics from 2019 [24]. Besides, the participation of hospital units with special interest in nutrition medicine is probably higher, and patients who are severely ill and thus not able to answer questions might not be well represented in the survey.

## Conclusion

In German hospital patients participating in the nutritionDay project from 2016 to 2020, recent and current low food intake (LI_RC_) was observed in every fifth patient overall, in every fourth gastroenterological but only every ninth neurological patient. Female sex, weight loss history, poor subjective health and functional status were related to LI_RC_ in the whole sample, and in several medical disciplines. Medical staff from all specialities, but from oncological and gastroenterological wards in particular, should assess food intake before hospitalisation at hospital admission and subsequently monitor food intake during the course of the hospital stay to intervene in time with nutritional therapy and further assessment of malnutrition. On top of this, our findings might help healthcare professionals in certain disciplines to focus on specific patient subgroups that are at high risk for LI_RC_ and therefore require additional nutritional treatment. In a next step, it should be evaluated whether nutritional therapy improves clinical outcome in these patient subgroups. Moreover, additional analyses of LI_RC_ in international samples and inclusion of dynamic variables such as the development of food intake are of further interest. Analysis of outcome data in a LI_RC_ patient group is desirable to better understand its role in comparison to current low intake only.

## Supplementary information

Table S1

Table S1 Text Summary

## Data Availability

The data here analysed was provided by nutritionDay worldwide (office@nutritionday.org). Data is available upon approval of a scientific project proposal by the nutritionDay worldwide scientific board and completion of a data sharing agreement. Additional data collection was enabled by the staff of the Klinikum Fürth, Germany.
