# Supplementary material for: Recent and current low food intake – prevalence and associated factors in hospital patients from different medical specialities
Source: Eur J Clin Nutr. 2022 Apr 11;76(10):1440–8. doi: 10.1038/s41430-022-01129-y (PMC9550619; doi:10.1038/s41430-022-01129-y)
Supplement: Supplementary file 2 — Table S1 Text Summary [file 41430_2022_1129_MOESM2_ESM.docx]

In Table S1, the univariate binary logistic regression models of recent and current low food intake are presented for the whole sample. All models with p-value below 0.1 were taken into account for a multivariate analysis, which was calculated in the total population and according to medical speciality.
